# Co-production of Diagnostic Excellence – Patients, Clinicians, and Artificial Intelligence: Comment on "Achieving Diagnostic Excellence: Roadmaps to Develop and Use Patient-Reported Measures With an Equity Lens"

**DOI:** 10.34172/ijhpm.8973

**Published:** 2025-06-17

**Authors:** Sumant R. Ranji, Benjamin I. Rosner

**Affiliations:** ^1^Division of Hospital Medicine, Department of Medicine, San Francisco General Hospital, San Francisco, CA, USA.; ^2^Division of Clinical Informatics and Digital Transformation, Department of Medicine, University of California San Francisco, San Francisco, CA, USA.; ^3^Division of Hospital Medicine, Department of Medicine, University of California San Francisco, San Francisco, CA, USA.

**Keywords:** Diagnostic Excellence, Patient-Reported Measures, Roadmaps

## Abstract

Patients often experience long journeys within the healthcare system before obtaining a diagnosis. Though progress has been made in measuring the quality of diagnosis, existing measures largely fail to capture the diagnostic process from the patient’s perspective. McDonald and colleagues’ paper presents 7 overarching goals for the use of patient-reported measures (PRMs) in diagnostic excellence and presents visual roadmaps to guide the development, implementation, and evaluation of these measures. To accelerate the real-world use of PRMs, organizations should initially prioritize the use of patient-reported metrics that are already in development, such as patient-reported experience measures. Pairing PRMs with artificial intelligence (AI) techniques, such as "diagnostic wayfinding" (a dynamic diagnostic refinement process that also includes analysis of electronic health record data and metadata to characterize the diagnostic journey), should also improve diagnostic performance. Ultimately, combining PRMs with technological advancements holds the potential to achieve true co-production of diagnostic excellence.

 When a patient develops a new health problem, the road to obtaining a diagnosis can be winding and unpredictable. Some progress has been made in measuring the quality of diagnosis, yet existing measures largely focus on measuring quality for specific diseases or within the context of a single clinical encounter. As an example, of the 17 diagnosis-related measures currently under consideration by the US Agency for Healthcare Research and Quality’s Quality Indicators Program, 16 are disease-specific metrics.^1^ While necessary, these measures may fail to capture the complexity of patients’ diagnostic odysseys, as they inherently measure diagnosis from the clinician’s perspective. To truly understand the impact of missed or delayed diagnoses on patients, it is necessary to understand the diagnostic process as experienced by patients themselves.^2^

 In this issue of the *International Journal of Health Policy and Management*, McDonald et al^3^ present 7 exemplar goals for patient-reported measures (PRMs) in diagnostic excellence and propose visual roadmaps as templates to guide the development, endorsement, implementation, evaluation, and real-world use of such measures. The goals seek to improve diagnosis from the patient perspective by establishing a safety net of diagnostic continuity, developing alert systems when patients are at risk of misdiagnosis, establishing organizational quality improvement, supporting diagnostic excellence research, evaluating routine screening, identifying and understanding geographic and demographic diagnostic disparities, and enabling patient storytelling of the diagnostic experience. By using an equity-focused human-centered design approach with active engagement of patients throughout the process, the authors ensure that the goals and roadmaps reflect the needs of patients most affected by inadequacies in the current diagnostic process. Equity is considered a cross-cutting principle applicable to each of the goals and roadmaps; within each roadmap, the authors provide information on facilitators and challenges to achieving each goal.

 McDonald and colleagues’ paper is an important step toward the goal of promoting patients as co-producers in diagnosis because it stitches together individual elements of patient reporting into an overall framework. In contrast to the traditional role of clinicians as diagnosticians, there is increasing recognition that patients have a crucial role to play in the diagnostic process – both as partners in establishing a diagnosis and ultimately assessing the diagnostic process. It will be impossible to achieve equity in diagnosis until diagnostic excellence is co-defined by patients and not just clinicians. Yet, as McDonald acknowledges, progress toward this goal will be measured in years or even decades – the time it will take to advance each PRM goal from development to implementation and practice change. How can this process be accelerated?

 In part, advancing PRMs into diagnosis can be accelerated by identifying those PRMs that are already in process and can enter the roadmap frameworks mid-stream. These include, for example, patient-reported outcomes and associated measures, and patient-reported experiences and associated measures.^4,5^ These can then be used to understand the diagnostic process itself. However, even when valid and reliable patient-reported outcome measures and patient-reported experience measures exist, a general lifecycle framework from development to implementation may not serve all health systems equally.^6^ Specifically, the challenges described in McDonald’s roadmap templates hint at the substantial system-level, service-level, and individual-level barriers to implementing PRMs, including, for example, organizational inertia, data collection burden, and health literacy and other social risk factors.

 Another means to potentially accelerate the process is the use of emerging generative artificial intelligence (AI). Although the explosion of this technology came about after the Expert Convening described in the paper, the opportunity for reciprocal benefit—AI accelerating collection and analysis of patient reporting, and patient reporting informing and humanizing AI—is not lost upon the authors. AI is now being widely used in the diagnostic process, particularly for specific diagnostic applications such as image analysis in radiology, pathology, ophthalmology, and dermatology.^7^ In addition, large language models (LLMs) such as ChatGPT have shown the ability to diagnose complex clinical scenarios, potentially serving as a decision support aid for clinicians.^8-10^ Patients are using LLMs as diagnostic aids as well. However, it is too early to know whether the risks previously known to be associated with self-diagnosis using online search engines^11,12^ will necessarily translate to self-diagnosis with LLMs,^13^ nor is it known how patient use of LLMs might be incorporated into the roadmapping approach described by McDonald et al. However, with the widespread adoption of “AI scribes” and related innovations, it is likely that some form of AI will be used in an ever-increasing proportion of clinical encounters. Achieving co-production of diagnostic excellence will therefore require integrating AI-based care innovations in roadmaps for implementation of PRMs.

 How could this be operationalized in practice? “Diagnostic wayfinding” offers an example of how advances in electronic health records and AI can be paired with PRMs to achieve co-production of diagnostic excellence.^14^ When a patient develops a new symptom, there is often a period of diagnostic uncertainty during which clinicians refine their diagnostic thinking and patients undergo diagnostic testing. At each step, new diagnostic information is acquired and integrated into diagnostic reasoning. While this process can take place in a single clinical encounter, patients may see multiple clinicians and undergo repeated rounds of diagnostic testing before a diagnosis is confirmed. In settings such as the United States, where the healthcare system is byzantine, this poses a considerable burden for patients and caregivers, especially for those who face barriers that limit their ability to engage with the healthcare system or must seek care in settings designed for rapid triage and stabilization rather than longitudinal care. As a result, many patients – especially those with rare or yet unnamed diseases, patients from historically marginalized populations, and patients with classically difficult-to-diagnose conditions – experience unacceptably long diagnostic odysseys. Diagnostic wayfinding seeks to support both patients and clinicians throughout the diagnostic journey by leveraging electronic health record data to characterize clinician and patient behavior throughout the diagnostic process—in essence, using “digital footprints” to interpret context and provide cues that guide the diagnostician.

 Digital diagnostic wayfinding can be paired with PRMs to improve diagnostic performance. For example, one goal of integrating PRMs into routine care is to develop real-time alerts when a patient is at risk of a diagnostic error. A PRM that indicates that the patient has not received an explanation for their health problem (such as via a patient survey) could be paired with diagnostic wayfinding data (such as natural language processing of patient portal messages or analysis of patterns of diagnostic testing) to alert the clinical team to the level of diagnostic uncertainty and the risk of diagnostic error. An AI tool could then interpret available data to provide context around the current diagnostic pathway and suggest guidance for additional diagnostic testing. By integrating information across time and setting, diagnostic wayfinding can also help identify patients who are only partially engaged with the health system—for example, patients who have multiple emergency department interactions for similar symptoms, or those who have been scheduled for but are unable to attend appointments or diagnostic testing. PRMs of health status can then be used to prioritize outreach and creative problem-solving to ensure such patients do not go undiagnosed.

 McDonald et al^3^ present an overarching framework for diagnostic excellence as a co-produced process between patients, caregivers, and clinicians. Leveraging existing validated PRMs and adding AI and wayfinding into this equation offer the potential to accelerate co-production by more fully characterizing patients’ diagnostic journeys and synthesizing diagnostic data from multiple sources to mitigate diagnostic errors. Amid the understandable excitement around the potential of AI to improve clinical care, it will be crucial to ensure that PRMs are at the center of how such tools are developed and integrated into clinical care.

## Ethical issues

 Not applicable.

## Conflicts of interest

 BIR and SRR receive grant support from the Gordon and Betty Moore Foundation for diagnostic excellence work. SRR receives grant support from the Agency for Healthcare Research and Quality for diagnostic excellence research projects.
